# Selective Control of the Apoptosis Signaling Network in Heterogeneous Cell Populations

**DOI:** 10.1371/journal.pone.0000547

**Published:** 2007-06-20

**Authors:** Diego Calzolari, Giovanni Paternostro, Patrick L. Harrington, Carlo Piermarocchi, Phillip M. Duxbury

**Affiliations:** 1 Burnham Institute for Medical Research, La Jolla, California, United States of America; 2 Physics and Astronomy Department, Michigan State University, East Lansing, Michigan, United States of America; Laboratory of Neurogenetics, National Institutes of Health, United States of America

## Abstract

**Background:**

Selective control in a population is the ability to control a member of the population while leaving the other members relatively unaffected. The concept of selective control is developed using cell death or apoptosis in heterogeneous cell populations as an example. Control of apoptosis is essential in a variety of therapeutic environments, including cancer where cancer cell death is a desired outcome and Alzheimer's disease where neuron survival is the desired outcome. However, in both cases these responses must occur with minimal response in other cells exposed to treatment; that is, the response must be selective.

**Methodology and Principal Findings:**

Apoptosis signaling in heterogeneous cells is described by an ensemble of gene networks with identical topology but different link strengths. Selective control depends on the statistics of signaling in the ensemble of networks, and we analyze the effects of superposition, non-linearity and feedback on these statistics. Parallel pathways promote normal statistics while series pathways promote skew distributions, which in the most extreme cases become log-normal. We also show that feedback and non-linearity can produce bimodal signaling statistics, as can discreteness and non-linearity. Two methods for optimizing selective control are presented. The first is an exhaustive search method and the second is a linear programming based approach. Though control of a single gene in the signaling network yields little selectivity, control of a few genes typically yields higher levels of selectivity. The statistics of gene combinations susceptible to selective control in heterogeneous apoptosis networks is studied and is used to identify general control strategies.

**Conclusions and Significance:**

We have explored two methods for the study of selectivity in cell populations. The first is an exhaustive search method limited to three node perturbations. The second is an effective linear model, based on interpolation of single node sensitivity, in which the selective combinations can be found by linear programming optimization. We found that selectivity is promoted by acting on the least sensitive nodes in the case of weak populations, while selective control of robust populations is optimized through perturbations of more sensitive nodes. High throughput experiments with heterogeneous cell lines could be designed in an analogous manner, with the further possibility of incorporating the selectivity optimization process into a closed-loop control system.

## Introduction

Living cells carry out their functions, such as working, reproducing and dying, by appropriate response to extracellular and intracellular inputs to a complex network of signaling pathways. Genes which code for the proteins in these pathways are controlled by regulatory proteins which up-regulate or down-regulate these genes, depending on inputs to the signaling network. The enormous effort currently directed at understanding signaling networks may be subdivided into two areas, firstly extracting faithful wiring diagrams for the networks and, secondly developing methods to understand and control the messages which pass through them. In this contribution we develop the concept of selective control in diverse cell populations, and introduce computational methods which optimize selectivity for a particular signal for a designated member of a cell population.

We introduce the concept of selective control or selectivity in cell populations as the requirement of finding a set of inputs which induce one member of the population to produce a desired response while ensuring that the remaining members of the population have a minimal response. In the case of apoptosis or cell death, which we use as an illustrative example, we consider a population of cells and seek methods to kill a selected member of the population while ensuring the survival of the others in the population. Control of apoptosis is essential in a variety of therapeutic environments including cancer where cancer cell death is a desired outcome[1]–[4] and Alzheimer's disease where neuron survival is the desired outcome[5]–[7]. However in both cases these responses must occur with minimal response in other cells exposed to treatment that is, the response must be selective.

Though striking progress is occuring in the extraction of networks using a range of experimental data[8], [9], knowledge of signaling networks remains predominantly at the level of topology rather than detailed knowledge of the rate constants and non-linear message passing which occurs in the networks. Models to distinguish between members of a population of cells, for example different cancer cells and different normal tissue types, require differences in message passing parameters and/or expression levels of the genes in the network. Here, the computational procedures for selectivity in cell populations are elucidated using heterogeneous populations, where members of the population are distinguished by having message-passing efficiencies drawn from homogeneous random distributions.

Models of message passing in gene networks range from binary models with discrete message passing rules[10]–[12] to non-linear ordinary differential equations[13] and to stochastic spatio-temporal models[14] which are simulated using partial differential equations or Monte Carlo methods. Questions of interest also vary greatly, from generic questions about the number of attractors and their stability in random networks[15]–[19] to modeling the detailed dynamics of gene concentrations in particular pathways[20]–[22], and to the cellular response such as control of flagellar rotation in bacteria responding to chemotaxis. Some of the tools developed for the analysis of metabolic networks, both dynamically and using steady state flux balance approaches, can be profitably extended to signaling networks[23], [24]. In the flux balance approach (FBA), the ouput of a cell may be optimized with respect to an objective function and subject to the constraints of flux balance at each node in the network.

Though there are conceptual similarities between the FBA and our approach, there are also critical differences. Instead of flux balance, we require message passing rules which describe how a gene responds to the state of its neighbors. To illustrate the effects of different rules, we use an important example from systems biology, the apoptosis network. In particular we discuss the statistics of death signals produced by continuum and discrete message passing rules in this network. We also develop the concept of selective control. Rather than optimizing the objective for one cell or metabolic network, as occurs in FBA, we seek to optimize the response of one cell in a population while minimizing the response of other cells in the population. Selective control is demonstrated using exhaustive search over drug combinations in discrete models and using an approximate linear programming approach. Though drugs affecting specifically every node of the apoptosis network are not yet available, this is a very active field of pharmacological research and it is probably one of the biological networks where this ideal situation, from the control point of view, is closest to reality[25]. Moreover, the network we use is probably still an incomplete representation of the apoptosis network, both for the topology and for the kinetic parameters. Nevertheless, several authors have shown that useful results can be obtained from partially characterized models of biological networks[26], [27].

## Results

### Statistics of signaling

**Figure 1:**
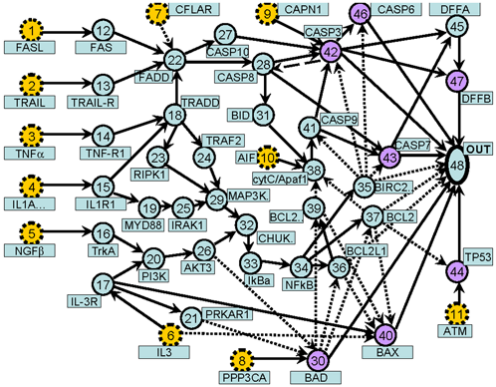
The human apoptosis network modified, as described in the text, from that in the Kegg database (hsa04210). There are 47 genes in the figure and an additional node which we label the output node. The 47 genes may be catagorized (see Table 1) as: input genes (dashed circles); membrane genes; death genes; life genes; and finally the remaining genes in the network. The output, or “death” node (48) is added to represent the cumulative effect of many genes implicated in the onset of the cell death machinery. In this figure, solid lines indicate promotion while short dashed links indicate inhibition. The long dashed link between CASP3 and CASP8 adds the possibility of feedback in the apoptosome.

We have modified the apoptosis network, hsa04210, of the Kegg database to that presented in Fig. 1, where network complexes consisting of several genes are split into individual nodes. Recent work emphasizes the importance of positive feedback between CASP3 and CASP8 denoted by the long dashed arrow in the figure[20], [22], though the importance of this feedback is not universally accepted. There are 47 genes in Figure 1 and an additional node which we label the output node. The 47 genes may be catagorized (see Table 1) as: input genes (dashed circles in the Fig. 1), which transmit signals to the network from the other parts of the cellular network; membrane genes which code for membrane proteins and complexes bound to membrane proteins; death genes which signal onset and execution of apoptosis; life genes which reduce apoptotic signals and are upregulated in many cancer cells; and finally the remaining genes in the network. The output, or “death” node (48 in Fig. 1) is added to represent the cumulative effect of many genes implicated in the onset of the cell death machinery.

**Table 1:** Typical roles of genes in the signaling network.

life	death	membrane
BIRC2..	BAD	FAS
BCL2L1	CASP6	FADD
BCL2	CASP3	TRADD
BCL2..	CASP7	TNF-R1
	BAX	RIPK1
	DFFB	TRAF2
	AIF	TRAIL-R
	TP53	IL1R1
		MYD88
		IRAK1
		NGFβ
		TrkA
		IL3
		IL-3R

We consider two types of models, those where the gene activities, *a_i_*, are continuous and those with discrete gene activities, *m_i_*. We use models with continuous activities to illustrate the generic statistics of signal propagation in the apoptosis network, while discrete models are more convenient for the exhaustive search methods used in the selectivity studies. In the discrete models the gene activity has discrete values up to a maximum value *M*, so that *m_l_* = 0,1…,*M*. Binary networks, where *M* = 1 have received the most attention, following the work of Kauffman[28]. We considered three discrete cases, *M* = 1,2,10, though here we focus upon M = 10 which is closer to the non-linear continuous behavior observed in experiment.

Each gene receives signals from the genes that it is connected to in the signaling network of Figure 1. The signal arriving at a gene depends on the strength of the connections to its neighbors in the network. We define the strength of these connections to be ω*_ij_* between the *i^th^* and *j^th^* genes. Since the network is directed, ω*_ij_*≠ω*_ji_*. The values of ω*_ij_* are poorly characterized even in metabolic networks where they correspond to reaction rates. In the absence of detailed knowledge about these connections we take them to be random variables and in this way develop a generic understanding of signal propagation in heterogeneous cell populations. The link weights, ω*_ij_*, have positive random values for promotion links and negative random values for inhibition links.

### Continuous models

In the continuous models, the edge weights ω*_ij_* are uniform continuous random variables and each gene has activity *a_i_* which is a continuous variable. The signal arriving at a gene is given by the sum,(1)
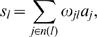
where *n*(*l*) is the set of genes which send signals to gene *l*. The signal *s_l_* arriving at gene *l* may be positive or negative, where a negative signal implies inhibition. However, the activity of a gene must be positive or zero so that a negative signal at a gene implies complete inhibition and the gene is switched off, so that its activity is set to zero. This is a basic non-linearity in signaling networks.

In addition, gene activity levels are often observed to depend in a non-linear way on the signals arriving at the gene. A common approximation to the nonlinear response in gene activity is the Hill equation[29],(2)
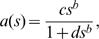
where *c*/*d* is the saturation value of the gene activity, *d* determines the onset of saturation and the exponent *b* is the cooperativity index. The case, *b* = 1, is Michaelis-Menten behavior characteristic of a chemical reaction in the presence of a substrate. The simplest case *b* = 1, *c* = 1, *d* = 0 is the majority rule signaling procedure, given by *a_l_* = *s_l_* for *s_l_* positive and *a_l_* = 0 for *s_l_* negative.

There are several procedures for simulating signal propagation through networks. In binary networks there has been considerable study of synchronous as opposed to asynchronous updates, where in the former case the gene activity levels at time *t* are used to update all of the activity levels at time *t*+1. In contrast asynchronous methods update gene activity randomly, for example one randomly chosen gene at a time, to model stochastic behavior. The number of attractors found in binary networks appears to depend on the update procedure 16]. In the absence of the feedback link between nodes 42 and 28 in Fig. 1, the network presented there has no loops. In this case signal propagation through the network is deterministic and non-chaotic. Furthermore signal propagation through the loopless network can be carried out in one sweep of the network by ordering the nodes according to their distance from the inputs. The nodes are then updated in order of their distance from the input, a procedure which we denote the optimal signaling algorithm(OSA). As described later, this procedure can be modified to take into account the feedback[20], [22] induced by the link between CASP3 and CASP8 in Fig. 1. In the absence of this link, the longest path from the inputs to the output has 15 links and hence the OSA algorithm is completed in 15 time steps.

We directly tested the effect of asynchronous, synchronous and OSA procedures on signaling in the loopless apoptosis network and found that they produce essentially the same results. This result is at first counterintuitive as undirected random networks show more complex behavior, such as chaos, and larger sets of attractors. However directed networks are deterministic so that for a given set of inputs in a network with fixed edge weights, and using deterministic message passing rules, there is a unique output. Statistical variations do occur however when the link weights are varied or stochastic noise is added to the message passing rules. Since we are interested in heterogeneous populations, which are analogous to quenched disorder, we consider variations in signaling due to variations in the edge weights.

**Figure 2:**
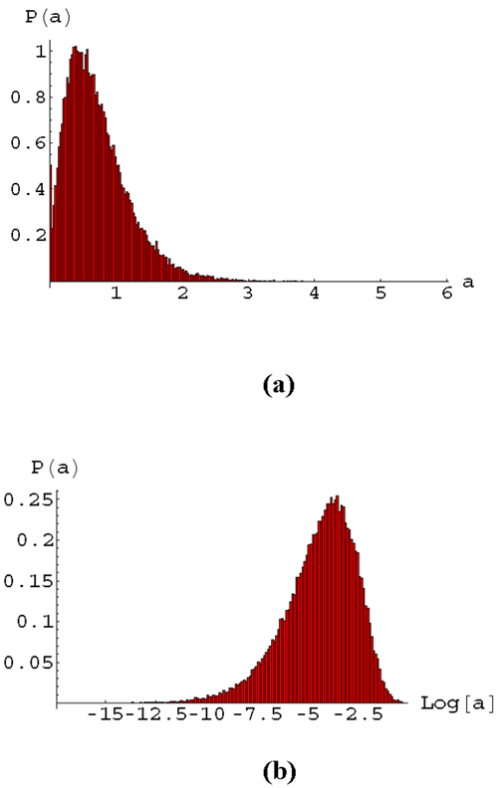
The distribution of gene activities in heterogeneous cell populations with a population size of 50,000. These distribution are for majority rule signaling with positive and continuous gene activities. a) The activity of the output node is close to a normal distribution due to many pathways arriving at the output node; b) Signal statistics at the gene NFkB, which is an internal node lying at the end of a chain of links (see Fig. 1), is close to log-normal. (see discussion in the text).

Typical statistical behavior of signals passing through the loopless apoptosis network are presented in Fig. 2 for an important gene, NFkB, in the interior of the network and also for the cumulative death signal at the output. These distributions are found by simulating 50000 different networks, where each network has links (values of ω*_ij_*) having weights drawn from a uniform distribution on the interval [0,1]. These simulations were carried out for a model with continuous activities *a_i_*, where Eq. (1) is used to find the total signal arriving at a gene and we use the linear relation *a_i_* = *s_i_* for positive signals and *a_i_* = 0 for negative signals. The input genes in the network were assigned random values on the interval [0,1]. Positive values of the signal arriving at the output node indicate cell death, while negative values denote life. Although the output node statistics (see Fig. 2a) is somewhat skew it is not too far from a normal distribution, however the statistics at NFkB (Fig. 2b) is highly skew and is almost lognormal. We now provide a simple explanation for the contrasting statistical behavior occurring for NFkB (34 in Fig. 1) and the output node (48 in Fig. 1).

**Figure 3:**
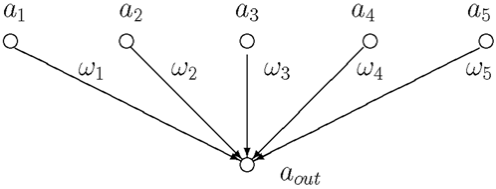
A parallel combination of signaling pathways with no series connections. For a large number of parallel connections, the output activity *a_out_* is normally distributed (see text).

**Figure 4:**
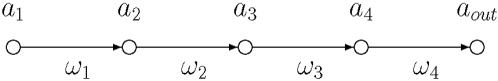
A signaling pathway with four steps in series and with no parallel connections. If the number of steps in the pathway is large, the ouput activity *a_out_* obeys log-normal statistics (see text).

Simplified models elucidating the origin of the signaling statistics observed in Fig. 2 are presented in Figs. 3 and 4. Many paths enter the death node (48) and this is simplified to a set of independent parallel paths in Fig. 3. According to Eq. (1), the death signal is then a sum of random variables and it is well known in that case that the statistics of the signal should be a normal distribution, in the asymptotic limit. The observed near normal behavior observed for the death node is then due to the fact that many parallel paths enter the death node. Deviations from the ideal normal distribution are expected for several reasons, including the fact that the activities cannot be negative, the presence of correlations in the signals entering the death node, and due to the fact that we are far from the asymptotic limit.

In contrast, NFkB is at the end of a chain of connections (see Fig. 1) and a simplified model of this connectivity is illustrated in Figure 4. In this case Eq. (1) yields,(3)
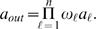
This is a random multiplicative process, so that if the link variables ω have random noise, then the output signal, *a_out_* asymptotically obeys log-normal statistics. The log normal distribution, in the variable *x*, is given by,(4)
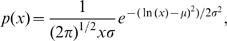
which is typically highly skew and exhibits large fluctuations. Here σ, μ are parameters in the distribution. The statistical variations of signals arriving at genes in complex networks clearly varies a great deal depending on the local connectivity of the genes. In cases where there are mostly linear pathways, as is believed to occur in some cancer cells, there is a greater potential for strong fluctuations in signaling statistics.

**Figure 5:**
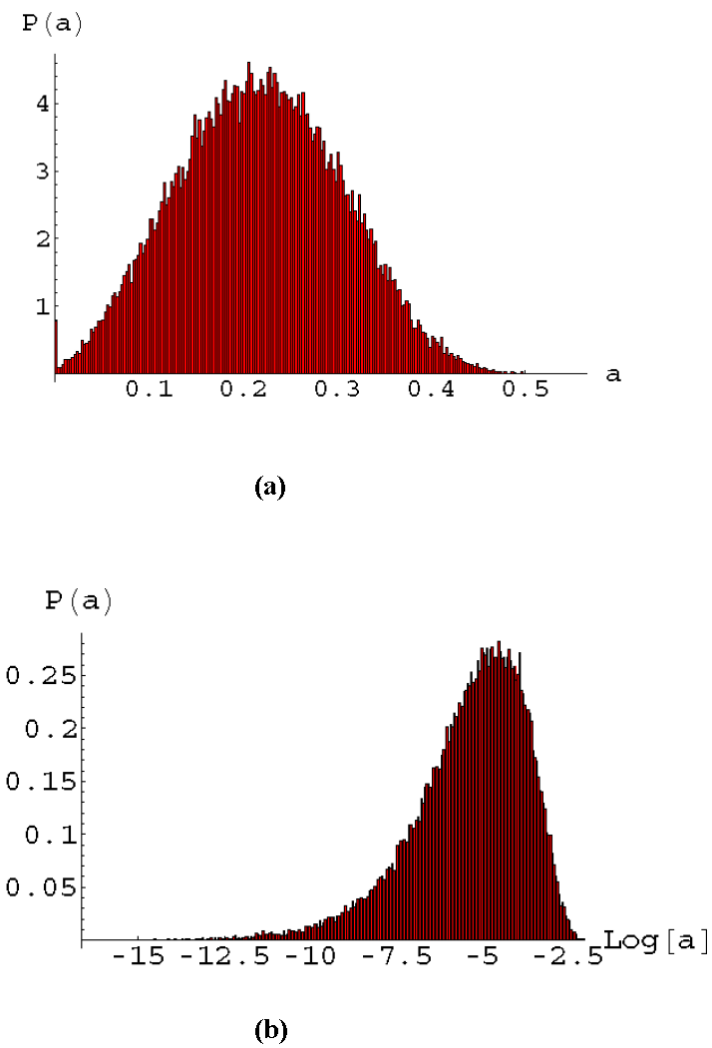
Majority rule signaling with non-linearity. The distribution of signals in heterogeneous cell populations with a population size of 50,000. These distribution are for majority rule signaling with positive, continuous gene activities calculated from the signal by using the Michaelis-Menten law *a* = *s*/(1+*s*). a) Signal statistics at the output node is similar to a normal distribution; b) Signal statistics at the gene NFkB remains close to log-normal despite the non-linear dependence of the activity on the signal.

Non-linearity is a hallmark of genetic response and we have studied the effect of a variety of non-linear activity-signal behaviors on signaling in the apoptosis network. We tested the effect of various Hill equation parameters on the activity statistics of the apoptosis network. In these calculations, each gene has the same non-linear behavior given by Eq. (2). We found that the generic behavior was similar to that presented in Fig. 2. One example, where we used the Michaelis-Menten limit of Eq. (2), is presented in Fig. 5. The statistics of the death node (Fig. 5a) remain close to a normal distribution, while the statistics of NFkB remains close to log-normal. The geometry of the network thus controls the signaling statistics even in the presence of non-linearity.

**Figure 6:**
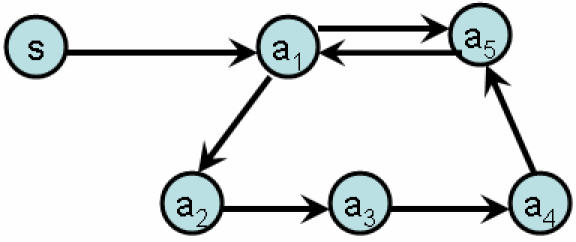
Feedback loop in the apoptosis network, following [20].

An important feature absent from the Kegg apoptosis network is feedback. The heavy dashed connection between CASP3 and CASP8 (nodes 42 and 28) produces feedback which has recently been found to be important in the apoptosome[20], [22]. This link leads to feedback as illustrated in the subgraph of Fig. 6. To elucidate the effect of feedback on signaling in the apoptosis network, we studied the response of the network in Fig. 6 with random weights on the edges and a range of signal strengths arriving at CASP8 (a_1_ in Fig. 6).

The equations for the signals, *s_i_*(*t*+1), arriving at time *t*+1 at the five genes in Fig. 6 are,(5)
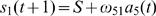

(6)
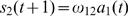

(7)
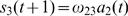

(8)
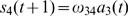

**Figure 7:**
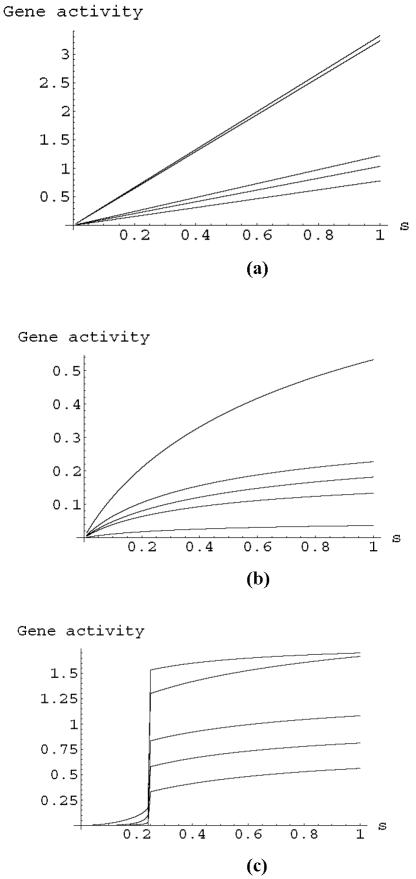
The steady state activity of genes in the feedback loop illustrated in Fig. 6. The top figure is the case of linear signaling (*d* = 0, *c* = *b* = 1 in Eq. (10)), the middle figure is for the Michaelis-Menten case (*b* = *c* = *d* = 1 in Eq. (10)), and the bottom figure was found using Eq. (10) with *c* = 2, *d* = 1 and *b* = 2 corresponding to co-operative non-linearity. In the top figure the link weights are ω_12_ = 0.367, ω_23_ = 0.864, ω_34_ = 0.754, ω_45_ = 0.617, ω_51_ = 0.718, ω_51_ = 0.828. In the middle and bottom figures the weights used were (0.417,0.847,0.287,0.456,0.614,0.521) and (1.812,1,207, 0.971,1.158, 1.924,1.489) respectively. The link weights were chosen to be random on the interval [0,*c*].

(9)
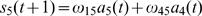
In this equation *S* is the input signal and ω*_ij_* is the strength of the signaling between nodes *i* and *j*. In heterogeneous population studies these links are taken to be random. The activity of each gene at time *t*+1 is found using a non-linear relation to the signal *s_i_*(*t*+1), given by the Hill equation,(10)
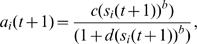
where *b*,*c*,*d* are model parameters. The typical activities of the five genes, *a_i_*(*t*), as a function of the input signal strength, *S*, are presented in Fig. 7 for three types of Hill equation parameters, linear (top figure), Michaelis-Menten (middle figure) and co-operative (bottom figure). As observed in modeling using ODE's[20], co-operative signaling leads to new behavior and a sharp onset of a transition between a low activity state and a high activity state. The behavior of Fig. 7c is typical of the co-operative case, and the location of the jump discontinuity and the values of the gene activities depend on the parameter values used in the simulations. One example is presented in this figure. We found that for each parameter set there is a steady state response at long times and this is the value that is plotted in the figures.

**Figure 8:**
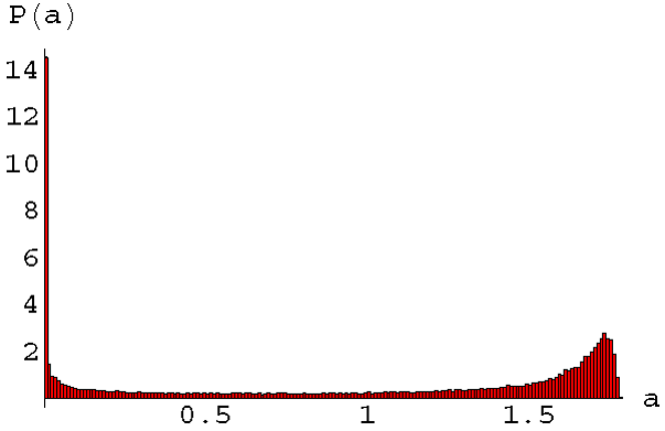
Death statistics in a heterogeneous population of 50,000 cells with co-operative non-linearity and feedback. The non-linearity parameters used in Eq. (10) were *c* = 1.8, *d* = 1, *b* = 2 and all of the link weights were chosen to be random on the interval [0,*c*].

We have also studied the effect of feedback on signaling statistics in heterogeneous cell populations using the full apoptosis network, Fig. 1. Signaling in this network is carried out by using the OSA procedure in combination with full iteration of the loopy sub-graph of Fig. 6. In the linear and Michaelis-Menten cases illustrated in the top two figures of Fig. 7, feedback amplifies the signal, but does not qualitatively change the statistics of death signals. However in the case of co-operative non-linearity where bi-stability and strong sensitivity to the input signal occurs (see Fig. 7c), the statistics of the death signal can become bimodal, as illustrated in Fig. 8. The signaling statistics can then be controlled by controlling the feedback and non-linearity in the network.

### Discrete models

Discrete models enable exhaustive search over the activity states of the genes and, as elucidated below, identification of the optimal combinations for selective control. In these models we discretize the weights and the activities into the same number of discrete levels, so that *m_i_* = 0,1,…*M* and |ω*_ij_*| = 1,…*M*, for a model where the activity level of a gene has a maximum integer value of *M*. We find that the generic behaviors for large values of *M* are similar to that of the continuous models of the last subsection. On the other hand, for binary networks where each gene has activity zero or one, the behavior is quite different, with a key novelty the fact that many genes have zero incoming signal and hence a decision must be made about their activity in this case. Using the momentum rule, where the state is maintained unless changed by an incoming signal, leads to a strong dependence on initial conditions as the initial state is unchanged unless a signal is received to change it. As in Eq. (1), the signal, *s_l_* arriving at gene *l* is a linear superposition given by,(11)
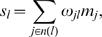
where *n*(*l*) is the set of genes which signal directly to gene *l*. In the discrete models, the signal, *s_l_*, produced by the superposition rule above is then normalised to *s_l_*/*n*(*l*) where *n*(*l*) is the number of neighbors of gene *l*. We use several different linear and non-linear relations to find the discrete activity of a gene, *m_l_*, from the normalized signal *s_l_*/*n*(*l*), as described below. In all cases, if the signal arriving at a gene is negative, the gene is completely inhibited and *m_l_* = 0, which is a basic non-linearity in both continuous and discrete signaling models.

**Figure 9:**
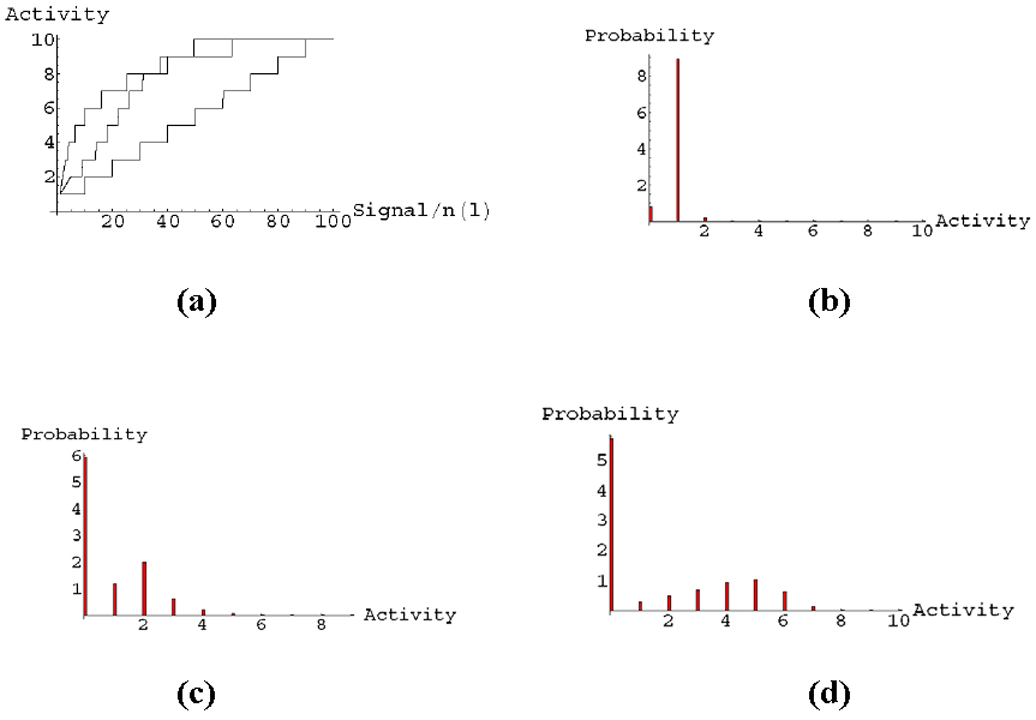
Discrete signal death statistics. a) Relations between the normalized signal, *s_l_*/*n*(*l*), arriving at a gene and it's discrete activity, from the top the behaviors are for the logarithmic function, Eq. (13), the sigmoidal function, Eq. (14) and the linear function, Eq. (12). The death statistics for these three models: b) linear function; c) sigmoidal function; d) logarithmic function. It is clear that the distribution of death activities becomes bimodal and broader as we go from the linear function to the logarithmic function.

In the linear rule, the discrete activity is found from the normalized signal using,(12)
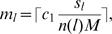
where *c*_1_ is a constant which we usually take to be *c*_1_ = 1. 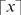 is the ceiling function, which raises a floating point number, *x*, to its next largest integer value. For the logarithmic rule the discrete activity is given by,(13)
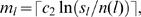
where the constant *c*_2_ is chosen so that the maximum signal corresponds to *m_l_* = *M*. In our case with *M* = 10, we take *c*_2_ = 2.17. The sigmoidal rule is given by,(14)
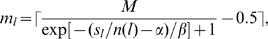
with the parameters α = 20 and β = 10. This has a form similar to the Fermi function in physics and to dose-response curves in radiation therapy. For *M* = 10 the maximum normalized signal which can arrive at a gene is 100. The functions (12–14) are constructed so that for small signals the gene activity is one, while signals of maximum value yield gene activity 10, ensuring that all possible relations between signal and activity are represented. These behaviors are presented in Fig. 9a.

The death statistics resulting from these discrete models are presented in Figs. 9b–d. The discrete linear model leads to unimodal statistics, however the sigmoidal and logarithmic functions lead to bimodal statistics. This is due to the fact that the latter functions amplify small signals, as is evident in Fig. 9a. In the next section we use these three discrete models in studies of selective control.

### Statistics of selective control

Selective control aims at changing the life/death signal of one member of a population with a minimal change of the remaining members of the population. We address in this section the question of how network topology and general signal propagation properties can be used to design strategies for selective control. The control of the life/death signal is realized by acting with external perturbations (drugs) on the nodes and on the signaling flow. As we have seen in the previous sections, the characteristics of the signaling through the network can be strongly dependent on the OSA rule. We will see below how strategies for selectivity are affected by these rules. General strategies for selectivity can be inferred by analyzing the statistics of the nodes involved in selective perturbations. For a given network topology, we can identify the nodes that are more likely to appear in a selective perturbation, and analyze their correlations. Important insight, such as the role of balancing pro-apoptosis and anti-apoptosis perturbations in selectivity, can be obtained from this analysis. Moreover, the correlation analysis revealed that for robust populations selective nodes tend to be the ones that produce the stronger change in the output signal. The opposite is true in the case of weak populations, for which selectivity is improved by acting on nodes that produce weak signal changes in the output.

### Exhaustive search in discrete models

In this subsection we carry out an exhaustive search of selective perturbations by discretizing the control parameters and signaling variables. In the next section, we will show how some of the key features of selectivity statistics can be captured with a simplified method based on linear programming optimization, which is less demanding from a computational point of view.

We start by generating a population of different apoptosis networks with the same topology, but random values for the initial gene expressions 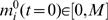 and random strength of the links ω*_ij_*∈[−*M*,−1] for inhibition, and *ω_ij_*∈[1,*M*] for stimulation (*M* = 10 in the numerical calculations). The chosen population needs to represent living cells, *i.e.* it must have the property(15)
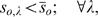
where *s̅* *_o_* is the signal life/death threshold value, λ is the index labeling individuals in the population, and *s_o_*_,λ_ is the output node signal. We take homeostasis into account by adding the constraint that each individual λ remains alive under fluctuations on the input nodes. After *s_o_*_,λ_ has been calculated for a given input, we let the input nodes fluctuate and we recalculate its value. If the output of a network is less than a threshold value, that is *s_o_*_,λ_<*s̅* *_o_*, for ten random fluctuations of the input nodes, then we keep the individual λ in the living population. In the analysis of selectivity, described below, a population of 100 was chosen as the number of different cell types in the human body is of this order. Preliminary studies indicate that, as expected, selective control is easier for smaller populations, provided the number of control nodes is fixed.

Once we have created a living population, we can start to study the effect of external perturbations on the nodes. These perturbations represent the effect of drugs that stimulate or inhibit one or more gene. We will therefore represent the effect of the drug by changing the gene expression levels in the nodes by δ*m_i_*. We will say that an individual λ̅ can be *selectively controlled* if we can find a perturbation on the gene expression levels δ*m_i_* with the property(16)
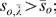

(17)
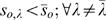
Though there are 48 nodes, 11 are input nodes and one is the output node, so there are 36 control nodes in the network. There are *M*−1 possible perturbations on each control node so the total number of possible perturbations on all the nodes, (*M*−1)^36^, which is too large to be explored exhaustively. Therefore, we considered perturbations that act only on *k*-subsets of all possible perturbations. For *k* = 1 this means that we are considering only single node perturbations, which requires a search over 36(*M*−1) possibilities. For *k* = 2 we are considering pairs of nodes, with 630(*M*−1)^2^ perturbation combinations. For an arbitrary *k*-subset the number of combinations is 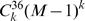.

In Table 2 we present the selectivity results for ten different populations with 100 individuals each and three different OSA rules. The columns in the Table 2 represent different *k*-subsets for the three different rules. The value for the threshold *s̅* *_o_* was set to 1. In the linear case, the average percentage of individuals that can be selectively killed within the *k* = 3 subset is about 63%. We have found that this average depends strongly on the value of the threshold, and it decreases for higher threshold values. For instance, by setting the threshold at *s̅* *_o_* = 7 the average selectivity (within the *k* = 3 manifold) is reduced to 8.3%. The logarithmic and sigmoid rules give a overall higher selectivity, with an average selectivity of 63.5% and 73.6%, respectively, for death threshold *s̅* *_o_* = 1.

**Table 2:** Number of individuals that can be selectively controlled in 10 populations with 100 individuals each.

Linear			Sigmoid			Logarithmic		
k = 1	k = 2	k = 3	k = 1	k = 2	k = 3	k = 1	k = 2	k = 3
4	22	58	1	32	76	9	30	64
2	25	61	4	35	71	9	34	62
2	21	67	4	31	77	9	28	64
3	36	68	1	37	66	10	29	58
2	30	68	3	28	74	8	32	63
2	39	71	2	33	74	9	34	62
1	26	53	3	37	76	4	30	68
1	28	60	5	31	66	10	33	63
1	28	66	3	37	76	4	30	68
1	23	57	5	37	80	10	33	63

The different columns refer to different *k*-subsets and different OSA rules, and with the death threshold *s̅* *_o_* = 1.

**Figure 10:**
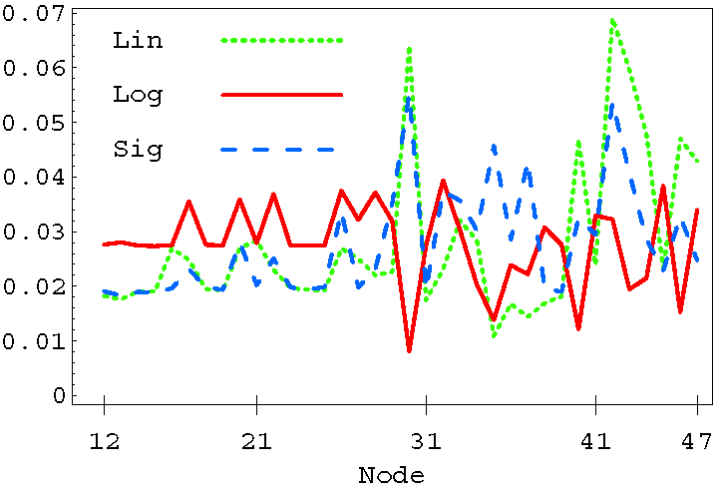
Distribution of nodes entering in selective combinations for the Linear (dotted), Sigmoid (dashed), and Logarithmic (solid) OSA rules.

We have studied the statistics of nodes entering in selective combinations. In the linear case, single-node selectivity is obtained by acting on BAD (30), IkBa (33), NFkB (34), BAX (40), CASP3 (42), CASP7 (43) or TP53 (44). These nodes can be divided into those that are pro- and anti-apoptosis, based on their average effect on the output signal. Single drug selective apoptosis is generally induced by stimulating a pro-apoptosis node, or by inhibiting an anti-apoptosis node. For instance, nodes IkBa (33) and NFkB (34) have on average an anti-apoptosis behavior, so they are mainly associated with negative δ*m*. The dotted line in Fig. 10 shows the distribution of nodes entering in all the selective combinations found using the linear OSA rule. Notice the peaks corresponding to the same nodes that can induce single-drug selectivity discussed above. In the figure, we also plot the distribution of nodes for the sigmoid (dashed line) and the logarithmic (solid line) rules. The sigmoid and the linear rule identify a very similar set of nodes that are more likely to enter in selective perturbations. Both BAD (30) and CASP3 (42) are strongly present in the linear and sigmoid model. The sigmoid rule slightly enhances the number of combinations in which the inhibition of anti-apoptosis genes represents the mechanism for selectivity, see e.g. CHUCK (32), IkBa (33), NFkB (34), BIRC2 (35) and BCL2 (37). Both linear and sigmoid rules suggest that selectivity might be better achieved by a direct up-regulation of caspases, or by acting on pro- or anti- apoptosis proteins of the BCL2 family. The logarithmic rule results in a different distribution, suggesting a different strategy for selectivity. Most of the selective combinations in the logarithmic case involve nodes upstream in the signaling network, indicating that the best strategy for selectivity is an action on cell-membrane FAS or TNF pathways.

**Figure 11:**
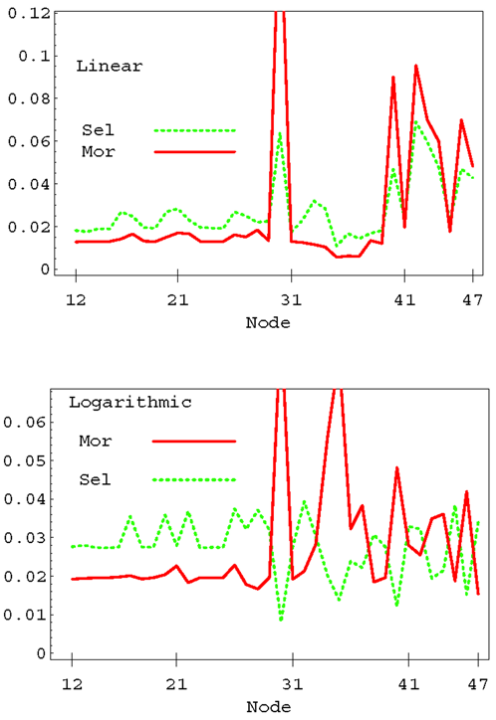
Distribution of nodes appearing in selective and mortal perturbations in the linear (top panel) and logarithmic (lower panel) OSA rules. The life/death threshold is set to *s̅* *_o_* = 1 for the two rules. The correlation between the mortality and selectivity distributions is 0.88 for the linear case and −.80 for logarithmic case.

Interesting correlations can be observed between the statistics of selective and mortal perturbations. Any perturbation that kills at least one member of the population is defined as *mortal*. Selective perturbations are mortal perturbations, but there are many more nonselective mortal combinations which kill more than one individual in the population. Fig. 11 shows the distribution of nodes entering in selective and mortal combinations in the case of linear (top panel) and logarithmic OSA rules (lower panel). The linear rule shows a strong correlation between nodes that appear in selective combinations and nodes that appear in mortal combinations. The correlation between the mortality and and selectivity distribution is 0.88. The logarithmic case is completely different (lower panel) and exhibits a strong anti-correlation between selectivity and mortality (−0.80). The anti-correlation in the logarithmic case can be reduced by increasing the threshold, and switches to correlation for values of the threshold larger than the average value of the output signal. This behavior can be understood in the following way. For a very high life/death threshold it is very difficult to find individuals that can be killed. In that case we can say that the population is very robust with respect to external perturbations. Therefore, if one individual can be killed, the perturbation that kills that individual is very likely to be selective. We have observed that by pushing the threshold to very high values the correlation between selectivity and mortality approaches one. The nodes that are involved in this case are the ones that are able to produce the strongest change in the output, and are the same nodes usually involved in many mortal combinations. On the other hand, if the population is weak, nodes that are highly mortal are likely to kill more than one individual at the same time, therefore selectivity is associated with nodes that are less mortal, i.e. those that produce small changes in the output signal. The robustness or weakness of a population is determined not only by the value of the life/death threshold, but also by the OSA rules giving different signaling statistics as discussed in the previous section. In fact, the behavior in Fig. 11 was obtained using the same threshold (*s̅* *_o_* = 1). There, the correlation/anticorrelation is a consequence of the higher sensitivity of the logarithmic rule to external perturbations, which implies that the population is much weaker compared to the linear case.

**Figure 12:**
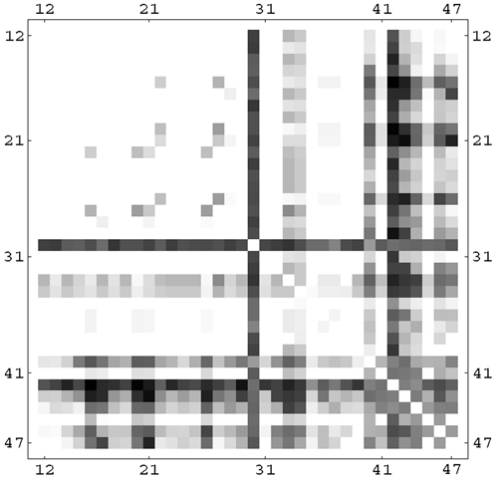
Correlation matrix for nodes appearing in the same selective perturbation in the linear OSA rule. Darker dots indicate that the two nodes given by the row and column of the matrix appear more often in the selective drug combinations.

**Figure 13:**
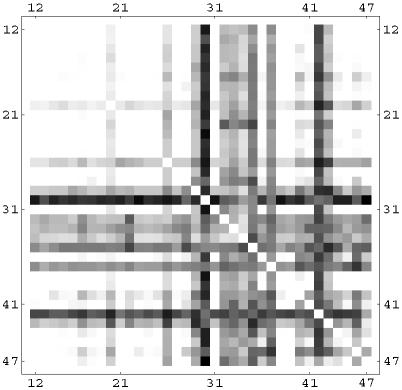
Correlation matrix for nodes appearing in the same selective perturbation in the sigmoidal OSA rule. Darker dots indicate that the two nodes given by the row and column of the matrix appear more often in the selective drug combinations.

**Figure 14:**
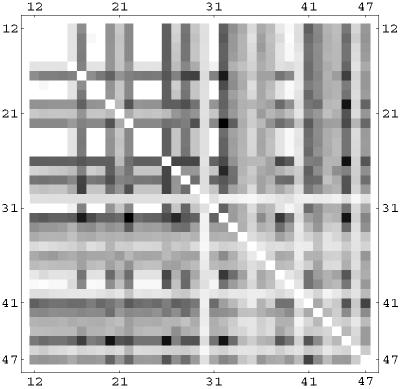
Correlation matrix for nodes appearing in the same selective perturbation in the logarithmic OSA rule. Darker dots indicate that the two nodes given by the row and column of the matrix appear more often in the selective drug combinations.

We have analyzed correlations between two nodes appearing in selective combinations. For the linear rule, this is shown in Fig. 12 using a correlation matrix plot. Darker dots indicate a higher number of selective combinations containing the two nodes given by the row and column of the matrix. Notice the strong presence of the mitochondrial BAD (30) and BAX (40), and the caspases CASP3 (42) and CASP7(43). However, these nodes often appear in combination with other nodes, many of which have an anti-apoptosis character. The balance of pro and anti-apoptosis perturbations is the key element which increases the selectivity from 1.5% in the *k* = 1 case to the 63% in the three-node perturbation. Notice also that in this linear case the nodes involved in selective combinations are often downstream (i.e. close to the output node) in the signaling network. We show in Fig. 13 and Fig. 14 the same correlation matrices in the case of the sigmoid and logarithmic OSA rules. In these models more nodes are involved in the selective combinations. The correlation pattern for the sigmoid rule shows strong similarities to the linear case. However, the logarithmic rule results in a qualitatively different pattern in the correlation matrix. Notice for instance that the role of BAD (30), which was dominant in the linear and sigmoid rule, is strongly reduced in this case. In contrast to the linear and sigmoid case, nodes in long pathways dominate in the selective combinations. Overall, the presence of nonlinearity in the OSA rules seems to enhance the possibility of selective control. This is also confirmed by the trend in the total number of *k* = 3 selective combinations, being 9×10^6^, 11×10^6^, and 26×10^6^ for the linear, sigmoid and logarithmic OSA rules, respectively.

### Linear programming methods

The exhaustive search method, discussed in the previous subsection, for selective combinations becomes very demanding when combinations with a large number of drugs are involved. A different approach consists in defining an effective model for the dependence of the output signal on the perturbations. The effective model is derived from the original OSA approach using a sensitivity analysis, and some of the selective perturbations found with the effective model are also selective perturbations for the original OSA problem. The advantage of the effective model is that selective combinations can be efficiently obtained by linear programming methods[30]. We will analyze below the statistics of the selective combinations for the linear OSA method obtained through the effective model. The form of the solutions in many cases involves a number of nodes larger than three, in contrast to the exhaustive method discussed in the previous section. The effective method therefore provides a different sampling of the full space of selective combinations. However, we will see that this different sampling leads to very a similar statistics for the selective nodes.

**Figure 15:**
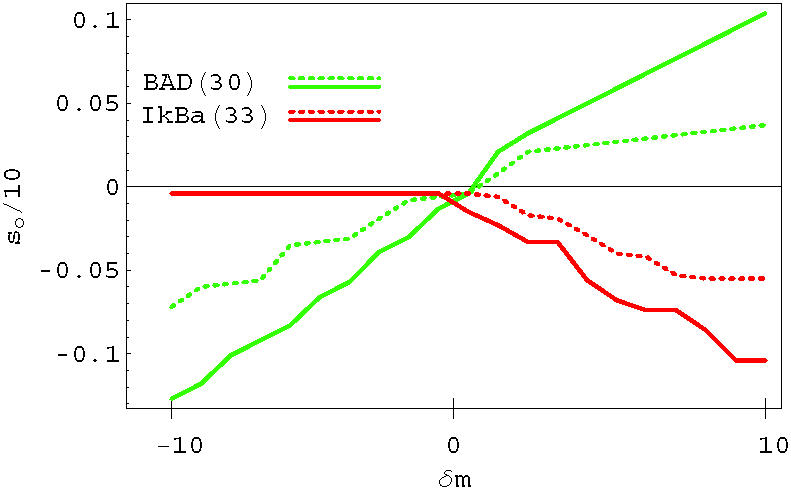
Output signal as a function of the perturbation strength on a pro-apoptosis (BAD, red increasing line) and anti-apoptosis node (IkBa, green decreasing line). The dashed line and the solid line refers to two different individuals.

The output signal from a OSA can be written as(18)
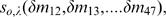
and depends in a nonlinear way on the perturbations of the internal nodes. A typical single node dependence is shown in Fig. 15 for a pro-apoptosis BAD (30) and an anti-apoptosis IkBa (33) node for two different individuals (solid and dashed lines) as a function of the strength of the single node perturbation. Notice that the single drug dependence follows a step-like dependence as a consequence of the discreteness of the gene expression levels. Moreover, due to the constraint that the activity must remain positive, there are regions in which the dependence on the external perturbation saturates. The effective linear model can be defined as(19)
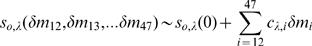
where the coefficient *c*_λ,*i*_ can be estimated by approximating the single drug dependence such as the ones in Fig. 15 with a linear dependence using interpolation methods. The linear interpolation works well only for some nodes and individuals, since in many cases the dependence is non-monotonic and highly nonlinear. Moreover, notice that we are neglecting higher order many-node effects that are included in the OSA approach. However, we will see below that this does not affect considerably the statistics of the genes that are more likely to appear in selective combinations. The interpolation method also allows us to identify nodes that lead to the highest variations of the output, and the selective combinations can be restricted to nodes within that set. The smallest coefficients *c*_λ,*i*_ can therefore be neglected. The reduction of the control parameter phase space by sensitivity analysis is often a key element in global optimization problems. This was shown explicitly in the case of parameter identification in biochemical reaction networks[31], [32]. Once the coefficient *c*_λ,*i*_ and the threshold value *s̅* *_o_* have been fixed, the selectivity problem can be recast in the form of a linear programming optimization problem where we minimize the cost function(20)
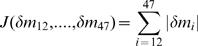
on the polytope defined by the constraint equations(21)
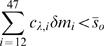

(22)
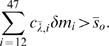
The solution provided by the linear programming method is optimal in the sense that it gives the global minimum of the function in Eq. (20) [30].

**Figure 16:**
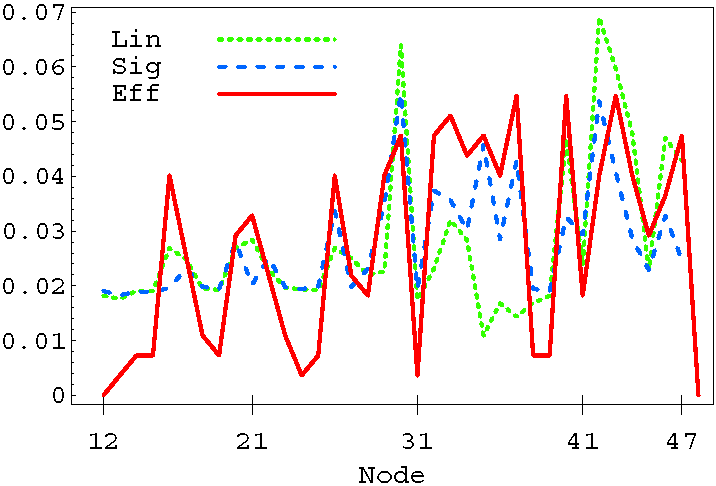
Distribution of nodes entering in selective combinations obtained with the Linear OSA (dotted), sigmoid (dashed) and with the effective linear programming method (solid line).

We show in Fig. 16 the statistics of the nodes found in the linear programming optimization (solid line) compared to the same statistics obtained with the exhaustive search described in the previous section (dotted line for linear, dashed line for sigmoid). The two approaches identify basically the same nodes as the ones that are more likely to be present in selective combinations. Notice the strong presence of BAD (30), BAX (40) and Caspases (42–44) in the three approaches. Also, the relatively strong peaks at IL-3R (17), PI3K (20), and AKT3 (26) are captured by the effective approach. These peaks suggest the possibility of selective control by acting on the AKT signaling pathway.

We also have used the optimal control parameter 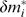 obtained with the linear programming on the original nonlinear OSA and checked the system for selectivity. Typically, we found that the linear programming solution is only partially selective for the original OSA rule. The drug combinations found from the linear programming method can selectively kill only 20% of the 100 individuals in the survivor population, which is considerably smaller than that found using exhaustive search. However, many selective combinations found by the linear programming approach are *quasi-selective*, in the sense that they kill two/three individuals in the population rather than one which is requirement for selectivity. This quasi-selectivity captured by the linear programming method is at the origin of the strong similarity in the distributions of Fig. 16.

## Discussion

Analysis of the statistical behavior of genes in the apoptosis network illustrates the importance of local connectivity on gene activity variations. Genes which receive signals from many parallel paths exhibit normal statistics while genes which lie at the end of a single pathway are prone to large statistical variations and highly skew statistics. Though non-linearity alone does not alter these broad conclusions, the combination of non-linearity and feedback may lead to bimodal statistics. This bimodality occurs due to combination of feedback and bistability[20], which in our signaling models is reflected in a sharp rise in activity with signal strength.

The concept of selective control in heterogeneous cell populations was developed, using the apoptosis network as an illustrative example. Selective control within a heterogeneous cell population is the ability to control one member of the population while leaving the other members relatively unaffected. General selective control strategies that are only dependent on the topology of the network and signaling rules can be inferred from analysis of networks with random link weights. For instance, linear and sigmoid rules identify the same set of nodes that are most efficient in selective control. These nodes can lead to a high degree of selectivity within a given population, especially by balancing pro- and anti- apoptosis perturbations. We have explored two methods for the study of selectivity. The first is an exhaustive search method limited to three node perturbations. The second is an effective linear model, based on interpolation of single node sensitivity, in which the selective combinations can be found by linear programming optimization. The two approaches identify the same strategies for selectivity. We have also identified a general rule that relates the life/death switching robustness of a population to the optimal selectivity strategy. Selectivity is promoted by acting on the least sensitive nodes in the case of weak populations, while selective control of robust populations is optimized through perturbations of more sensitive nodes. More generally selective control is a computational challenge in a broad range of systems biology problems where intervention needs to be directed at subsets of a diverse population.

At a more practical level, high throughput experiments with heterogeneous cell lines could be designed in such a way that the selectivity optimization process is part of a closed-loop control system[33]. Single drug measurements could be used to obtain directly the sensitivity in a given heterogeneous cell population. The linear programming optimization method can then use the sensitivity measurements to identify selective combinations in a model free way. The sensitivity/selectivity optimization can then be improved through iterated experiments in which various computational algorithms and experimental protocols are integrated.

## Methods

Two programs were used to generate the signaling statistics and to analyse selectivity. One procedure was written in Mathematica and the other in C++. These independent programs were used to check the accuracy of the numerical analysis of the signaling procedures and the signaling statistics.

The software used to run the exhaustive search tests is written in C++ and compiled for both the Linux 32 bit and 64 bit operating systems. We used two clusters, available at the Burnham Institute for Medical Research, Falcon and Bsrc. Both of them are Portable Batch System (PBS) clusters, so that they have a native implementation of queues management enabling submission of the same program multiple times to achieve virtual parallelism. Falcon has 128 CPUs organized in 64 nodes and each of them is a x86 32 bit 2.4 GHz with 1 GB of private memory; Bsrc has 128 CPU organized in 32 nodes and each of them is a x86 64 bit 2.0 GHz with 2 GB of private memory. Exhaustive search requires an exponential computational time in the number of nodes and projecting from the time needed for combinations of 1,2,3 nodes, that is respectively 1 minute, 1.5 hours and 7.5 hours, we estimate that around 90 days would be needed to study all the combinations of 4 drugs. The calculations on the continuous models and the linear programming optimization were implemented using Mathematica on a personal computer. The NMinimize function in Mathematica finds the global minimum when the objective function and constraints are linear.
